# Hybrid Fuzzy Clustering Method Based on FCM and Enhanced Logarithmical PSO (ELPSO)

**DOI:** 10.1155/2020/1386839

**Published:** 2020-03-18

**Authors:** Jian Zhang, Zongheng Ma

**Affiliations:** School of Mechanical Engineering, Tongji University, Shanghai 200092, China

## Abstract

Fuzzy c-means (FCM) is one of the best-known clustering methods to organize the wide variety of datasets automatically and acquire accurate classification, but it has a tendency to fall into local minima. For overcoming these weaknesses, some methods that hybridize PSO and FCM for clustering have been proposed in the literature, and it is demonstrated that these hybrid methods have an improved accuracy over traditional partition clustering approaches, whereas PSO-based clustering methods have poor execution time in comparison to partitional clustering techniques, and the current PSO algorithms require tuning a range of parameters before they are able to find good solutions. Therefore, this paper introduces a hybrid method for fuzzy clustering, named FCM-ELPSO, which aim to deal with these shortcomings. It combines FCM with an improved version of PSO, called ELPSO, which adopts a new enhanced logarithmic inertia weight strategy to provide better balance between exploration and exploitation. This new hybrid method uses PBM(F) index and the objective function value as cluster validity indexes to evaluate the clustering effect. To verify the effectiveness of the algorithm, two types of experiments are performed, including PSO clustering and hybrid clustering. Experiments show that the proposed approach significantly improves convergence speed and the clustering effect.

## 1. Introduction

In order to obtain effective information on huge quantities of data quickly and accurately, many methods have been proposed. As an unsupervised learning method, clustering analysis is one of the vital means in dealing with these data whose objective is to partition an unlabeled dataset into a number of clusters, such that elements in same cluster show a high level of similarity, while elements from different clusters show a high level of dissimilarity. The clustering technique has been studied extensively in a variety of application fields such as data mining, machine learning, pattern recognition, and image segmentation [1–3].

Clustering algorithms can be further divided into two basic categories: hard and fuzzy [4]. Hard clustering methods assign each object to a single group, while fuzzy clustering methods introduce membership degrees between objects and the different clusters of the dataset and assign each element of a dataset to multiple clusters simultaneously in accordance with the membership function matrix. Therefore, the latter can handle overlapping partitions.

The most popular fuzzy clustering algorithm is fuzzy c-means (FCM) which was proposed by Bezdek et al. [5] and has been widely used in multiple domains [6, 7]. The goal of FCM is to minimize the criterion function and obtain a more accurate membership matrix gradually. But the random selection of center points makes iterative process fall into the saddle points or local optimal solution easily. Furthermore, if the datasets contain severe noise points or if the datasets are high dimensional, such as bioinformatics [8], the alternating optimization often fails to find the global optimum.

However, these shortcomings have motivated the proposal of alternative approaches for fuzzy clustering, many of which are extensions of FCM. A kernel-based FCM (KFCM) was proposed by Zhang and Chen [9], which replaces the Euclidean distance metric with a kernel metric to achieve better mapping for nonlinear separable datasets. Lin [10] proposed a novel evolutionary kernel intuitionistic FCM clustering algorithm (EKIFCM) that combines intuitionistic fuzzy sets (IFSs) with KFCM and utilizes genetic algorithms (GA) to optimize the parameters of EKIFCM simultaneously. Although these FCM versions aim to achieve good performance in fuzzy clustering, they do not improve the random initialization process of FCM and still fall into local optimum easily [11].

The probability of finding the global optimum may be increased by stochastic methods such as evolutionary or metaheuristic optimization algorithms. As one of the most famous metaheuristic methods, PSO has become one of the most popular metaheuristics and an important tool for many applications due to its versatility and simplicity, and it is found that it can provide better initial centroids for the FCM algorithm to improve the FCM results, and thus this has motivated the proposal of many PSO-based methods for hard clustering [12] and some PSO-based methods for fuzzy clustering [11, 13, 14]. Cura [15] presented a new PSO approach to the clustering problem, employing the pure PSO technique to solve both clustering problems with both known and unknown numbers of clusters, which provides a new idea for clustering.

Izakian and Abraham [16] proposed a hybrid fuzzy clustering method based on FCM and fuzzy PSO (FPSO), and their experiments show better results than FPSO and FCM. The quantum-behaved particle swarm optimization (QPSO) with a fully connected topology is coupled with the FCM, forming a new version of hybrid method called QPSO-FCM [17]. However, these PSO-based methods are much slower compared to the traditional methods which may limit their practical applications.

Another problem with PSO-based clustering methods, according to Alam [12], is the need to tune a range of parameters before they are able to find a better solution. For overcoming these shortcomings, hybrid methods for fuzzy clustering based on fuzzy c-means and improved particle swarm optimization (FCM-IDPSO) were proposed by Silva Filho et al. [18], who introduced the IDPSO for adjusting the parameters dynamically during training and tackling the two main problems of PSO-based clustering methods. Many improved PSO-FCM clustering methods have been successfully applied to practical applications [19–22]. It is worth noting that the complex structure of PSO-based methods and the huge amount of computation make the algorithm have room for further improvement.

In recent years, many excellent hybrid methods have been proposed for optimal cluster analysis, which do not use PSO as optimization algorithm, such as CRO-FCM [23] which uses chemical-based metaheuristic obtaining optimal cluster centers for FCM; ETLBO-FCM [24] incorporates elicit teaching learning-based optimization and FCM to overcome the major limitations of FCM; Rahul et al. [25] introduced bat optimization to FCM and utilized maxi-min classifier to determine the count of clusters, and the results showed that the clustering accuracy is improved. These studies have greatly promoted the development of clustering algorithms.

One of the main contributions of this paper is to introduce a new version PSO with enhanced logarithmic decreasing strategy (ELPSO) for clustering. Based on this strategy, ELPSO takes different inertia weight values during various periods adaptively and thus provides better balance between exploration and exploitation and avoids falling into local minima quickly, thereby obtaining better solutions. The other contribution of this paper is to propose a new method for the fuzzy clustering problem using hybridization combining FCM with ELPSO, named FCM-ELPSO, which makes use of the merits of both algorithms. This hybrid method introduces ELPSO for training process and uses ELPSO's global exploration to find a suitable initial clustering prototype for FCM and the local exploration to avoid falling into local optimum and utilizes the fast convergence of FCM to improve the results and convergence time. Both clustering methods are tested based on UCI datasets independently, and the results are compared to other PSO-based clustering methods, respectively.

The structure of the paper is as follows.  Section 2 outlines all necessary prerequisites. In Section 3, a new version of PSO for clustering, named ELPSO, and the hybrid method (ELPSO-FCM) are proposed.  Section 4 includes the results of experiments based on UCI datasets. In Section 5, main conclusions are covered.

## 2. Theoretical Basis

In this section, we briefly describe some basic concepts of FCM, original PSO (or standard PSO, SPSO) and some improved versions of PSO with different inertia weight strategies, and a cluster index which is used in the hybrid method for evaluating the clustering effect.

### 2.1. FCM

We define *S*={*s*_1_,…, *s*_*j*_,…, *s*_*N*_} as a clustering dataset of *N* objects indexed by *j*; each object *s*_*j*_ is represented by a vector of quantitative variable. We define *B*={*β*_1_,…, *β*_*i*_,…, *β*_*C*_} as the prototypes of *C* clusters listed by *i* and *U*=[*u*_*ij*_]_*C*×*N*_ as a fuzzy partition matrix, where *u*_*ij*_ indicates the membership of the *j*^th^ object with the *i*^th^ prototype. *s*_*j*_, *β*_*i*_ ∈ *R*^*Q*^, where *Q* is the data dimensionality. The constraints on *u*_*ij*_ are as follows:

(1) $$\begin{array}{c} u_{ij}\in \left[0,1\right],\;\forall i=1,2,\ldots ,C;\;\forall j=1,2,\ldots ,N; \end{array}$$

(2) $$\begin{array}{c} \sum_{i=1}^{C}u_{ij}=1,\;\forall l=1,2,\ldots ,N; \end{array}$$

(3) $$\begin{array}{c} 0<\sum_{j=1}^{N}u_{ij}<N,\;\forall i=1,2,\ldots ,C. \end{array}$$

The goal of the FCM algorithm is to find the optimal prototype matrix and the corresponding membership degree matrix that minimize an objective function given by the following equation:

(4) $$\begin{array}{c} J=\sum_{i=1}^{C}\sum_{j=1}^{N}{\left(u_{ij}\right)}^{m}d_{ij}^{2}, \end{array}$$

where *m*(*m* > 1.0) is the fuzzy weighting exponent and *d*_*ij*_ is the Euclidean distance that indicates the dissimilarity from data vectors *s*_*j*_ to cluster center *β*_*i*_.

The parameter *d*_*ij*_ is obtained by the following equation:

(5) $$\begin{array}{c} d_{ij}=\left\|s_{j}-\beta_{i}\right\|. \end{array}$$

To minimize the criterion *J*, the clustering prototypes *β*_*i*_ and the membership degrees *u*_*ij*_ are updated according to equations (6) and (7), respectively.

(6) $$\begin{array}{c} \beta_{i}=\frac{\sum_{j=1}^{N}{\left(u_{ij}\right)}^{m}s_{j}}{\sum_{j=1}^{N}{\left(u_{ij}\right)}^{m}}, \end{array}$$

(7) $$\begin{array}{c} u_{ij}=\frac{1}{{\sum_{k=1}^{C}\left(d_{ji}/d_{ki}\right)}^{\left(2/\left(m-1\right)\right)}}. \end{array}$$

After computing the memberships of all the objects, the new prototypes of the clusters are calculated. The process stops when the prototypes stabilize. That is, the prototypes from the previous iteration are of close proximity to those generated in the current iteration, normally less than an error threshold.

### 2.2. Original Particle Swarm Optimization

PSO is originally introduced in terms of social and cognitive behavior of bird flocking and fish schooling. The potential solutions are called particles which fly through the problem space by following the current best particles. Each particle keeps track of its coordinates in the problem space which are associated with the best solution that has been achieved so far. The solution is evaluated by the fitness value, which is also stored. This value is called *pbest*. Another best value that is tracked by the PSO is the best value, obtained so far by any particle in the swarm. The best value is a global best and is called *gbest*. The search for the better positions follows the rule as equations (8) and (9):

(8) $$\begin{array}{c} v_{l}\left(t+1\right)=\omega \left(t\right)v_{l}\left(t\right)+c_{1}r_{1}\left(pbest_{l}\left(t\right)-x_{l}\left(t\right)\right)+c_{2}r_{2}\left(gbest\left(t\right)-x_{l}\left(t\right)\right), \end{array}$$

(9) $$\begin{array}{c} x_{l}\left(t+1\right)=x_{l}\left(t\right)+v\left(t\right), \end{array}$$

where *x*_*l*_ and *v*_*l*_ are position and velocity vector of the particle *l*, respectively; *ω* is the inertia weight; *c*_1_ and *c*_2_ are positive constants, called acceleration coefficients which control the influence of *pbest*_*l*_ and *gbest* in search process; and *r*_1_ and *r*_2_ are random values in the range [0, 1]. The fitness value of each particle's position is determined by a fitness function, and PSO is usually executed with repeated application of (8) and (9) until a specified number of iterations have been exceeded or the velocity updates are close to zero over a number of iterations.

### 2.3. Some Improved Versions of PSO with Different Inertia Weight Strategies

Using statistical theory to analyze the variance of the basic parameters of PSO, including inertia weight and accelerating constants, it can be considered that the inertia weight has tremendous impact on the overall performance of PSO [26]. Many studies have shown that larger inertia weight values have better global search capabilities, and smaller inertia weight values have advantages in local exploitation [27]. So, different adaptive strategies of inertia weight are proposed to achieve a better balance between exploration capabilities and development capabilities and get more stable and satisfactory results, such as linear, nonlinear, fuzzy rules, random, and other strategy-based inertia weights.

In this section, three kinds of inertia weight strategies will be emphatically reviewed which are widely used in a variety of application domains, and the process of corresponding algorithms can be found in [28–30]. The method proposed in this paper will be compared with the above algorithm in Section 4.

#### 2.3.1. Linear Inertia Weight Strategy

The monotonic decreasing inertia weight adjustment strategy is introduced into PSO by Eberhart [28] and aimed to enhancing the fine-tuning ability of PSO. But linear inertia weight strategy cannot achieve the accurate balance between local search and global search due to the nonlinearity and complexity of the PSO search process. So, it does not always perform better than an appropriate fixed inertia weight when the inertia weight decreases gradually as the iteration proceeds.

#### 2.3.2. Natural Exponential Inertia Weight Strategy

Inspired by linear decreasing inertia weight strategy, Chen et al. [29] proposed two inertia weight strategies of natural exponential functions. Based on their experimental settings, these natural exponential strategies have a faster convergence speed in the early stage of PSO search process compared with the linear adjustment strategy.

#### 2.3.3. Random Inertia Weight Strategy

It is difficult to predict whether in a given time the exploration or exploitation would be better in the dynamic environment. So, randomness is introduced into the inertia weight strategy of PSO to address this problem in [30]. Using particle swarms to track and optimize dynamic systems, a new way of calculating the inertia weight value is proposed.

### 2.4. Cluster Index PBM(F)

Pakhira et al. [31] proposed a validity index called the PBM index. The index is developed for both crisp and fuzzy clustering; however, here we review only the fuzzy version of the index called the PBM(F) index. The index is defined as

(10) $$\begin{array}{c} V_{\text{PBMF}}={\left(\frac{1}{C}\times \frac{E_{1}}{J_{m}}\times D_{C}\right)}^{2}, \end{array}$$

where $E_{1}=\sum_{j=1}^{N}u_{ij}\left\|s_{j}-\dot{\beta }\right\|$; *D*_*C*_=max_*i*,*n*=1_^*C*^‖*β*_*i*_ − *β*_*n*_‖; *C* is the number of clusters; and $\dot{\beta }$ is the center of dataset *S*.

*J*_*m*_ is different from *J* and considered to be

(11) $$\begin{array}{c} J_{m}\left(U,Z\right)=\sum_{j=1}^{N}\sum_{i=1}^{C}{\left(u_{ij}\right)}^{m_{1}}\left\|s_{j}-\beta_{i}\right\|, \end{array}$$

where *N* is the total number of patterns in the dataset, *U*(*S*)=[*u*_*ij*_]_*C*×*N*_ is a partition matrix for the data, and *β*_*i*_ is the centroid of the *i*^th^ cluster; here, the fuzzy parameter *m*_1_ is set to 1.5.

The factor, *E*_1_/*J*_*m*_, includes the sum of weighted intracluster distances for the complete dataset taken as a single cluster and that for the *c* cluster system. This factor is a measure of the compactness of a *C* cluster system. The factor *D*_*C*_ is the maximum intercluster separation in a *C* cluster system. This factor signifies between cluster separation. Higher values of the PBM(F) index indicate better clustering in the sense that the clusters are well separated and relatively compact.

## 3. Proposed Algorithms

In this section, we will introduce the new version PSO with enhanced logarithmic decreasing strategy in detail, named ELPSO, and give the algorithmic process for clustering application; next, based on ELPSO and FCM, a hybrid algorithm is formed for combining the merits of these two algorithms, called FCM-ELPSO.

### 3.1. Enhanced Logarithmical PSO (ELPSO)

In order to adjust the performance of particle swarm and balance the global search and local search capabilities of the swarm in flight process, a simple and effective inertia weight adjustment strategy was introduced into PSO and a new version of PSO, called enhanced logarithmic decreasing PSO (ELPSO), was developed. The new strategy function is formulated as follows:

(12) $$\begin{array}{c} \omega_{l}\left(t\right)={\left(\ln\left(2.1+t\right)\right)}^{\wedge }\left(-z\right), \end{array}$$

where *t* is the current iteration and *z* is the regulatory factor for fine-tuning ability of PSO, whose value can be set to 1.05 by experience. Equations (13) and (14) show the new velocity formula and position formula of particle *l* at instant *t* using the new inertia weights:

(13) $$\begin{array}{c} V_{l}\left(t+1\right)=\omega \left(t\right)V_{l}\left(t\right)+c_{1}R_{1l}\left(pbest_{l}\left(t\right)-X_{l}\left(t\right)\right)+c_{2}R_{2l}\left(gbest\left(t\right)-X_{l}\left(t\right)\right), \end{array}$$

(14) $$\begin{array}{c} X_{l}\left(t+1\right)=X_{l}\left(t\right)⊕V_{l}\left(t\right). \end{array}$$

The size of each element is consistent in equations (13) and (14) except the parameters *R*_1*l*_ and *R*_2*l*_. In order to increase the randomness of particle swarm search, we set the random value *R* as a matrix. Random matrixes of each particle will be initialized during every iteration, and the range of each element in the matrix is [0, 1].

Here, we give the method for clustering which employs the pure ELPSO technique.

Let the position of particle *l*, represented by *X*_*l*_, be the prototype matrix, whose size is *C* × *Q*, where *C* is the right cluster number and *Q* is the dimension of the datasets *l* ∈ [1, *P*] in which *P* is the size of population. In this way, *X*_*l*_ may be expressed as follows:

(15) $$\begin{array}{c} X_{l}=\left[\begin{array}{ccc} \beta_{11} & \cdots & \beta_{1Q}\\ \vdots & \ddots & \vdots \\ \beta_{C1} & \cdots & \beta_{CQ} \end{array}\right]. \end{array}$$

Therefore, a swarm represents a number of candidates' cluster center for the data vector. Each data vector belongs to a cluster according to its membership function and thus a fuzzy membership is assigned to each data vector. Each cluster has a cluster center per iteration and presents a solution which gives a vector of cluster centers. This method determines the position vector *X*_*l*_ for every particle, updates it, and then changes the position of cluster center. And the fitness function for evaluating the generalized solutions is stated as

(16) $$\begin{array}{c} f\left(X_{l}\right)=\frac{1}{J\left(X_{l}\right)}, \end{array}$$

where *J*(*X*_*l*_) is the objective function of FCM, as shown in equation (4), calculated for particle *l*. The smaller *J*(*X*_*l*_) is, the better is the clustering effect and the higher is the fitness function *f*(*X*_*l*_).

The fake code is shown as follows.

### 3.2. The Hybrid Methods for Fuzzy Clustering Based on Fuzzy c-Means and Improved Particle Swarm Optimization

Although FCM requires fewer function evaluations, it usually falls into local optima. In this section, the FCM algorithm is integrated with ELPSO algorithm to form a hybrid clustering algorithm called FCM-ELPSO which maintains the merits of both FCM and ELPSO algorithms. This hybrid method introduces ELPSO for training process and uses ELPSO's global exploration to find a suitable initial clustering prototype for FCM and the local exploration to avoid falling into local optimum and utilizes the fast convergence of FCM to improve the results and convergence time.

The fake code is shown as follows.

## 4. Experiments and Results

This section is divided into two parts: ELPSO clustering and hybrid clustering, can use Algorithm 1 and Algorithm 2 for obtaining corresponding results separately. All experiments are based on the platform Matlab 2016b and executed on an Intel core i7-8750H 2.20 GHz computer running Microsoft Windows 10.

For evaluating the performance of the proposed algorithms, nine well-known UCI Machine Learning Repository datasets have been selected: Abalone, Ecoli, Glass, Image segmentation, Page blocks classification, Spectf, Steel plates faults, Ultrasonic flowmeter diagnostics, and Yeast. These datasets include examples of low, medium, and high dimensional data with various partitions. A detailed description of the datasets is shown in Table 1.

### 4.1. ELPSO Clustering

The ELPSO, original PSO, and three improved versions with different inertia weight strategies shown in Section 2.3 will be tested here for evaluating the performance of these heuristic algorithms. Based on Abalone, Ecoli, Glass, and Image segmentation datasets, each method runs 30 times independently and total 500 iterations within every time.

**Figure 1:**
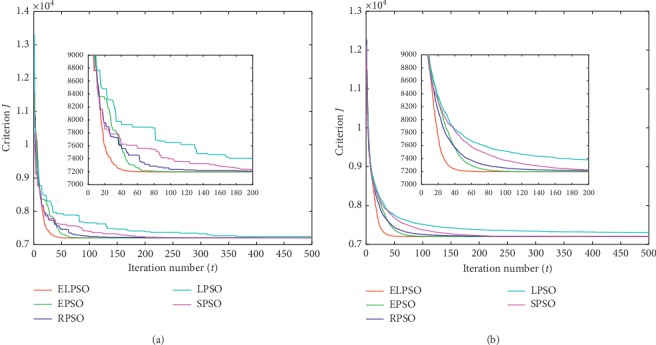
The clustering results of Abalone dataset. (a) The best result in 30 times. (b) Average result in 30 times.

**Figure 2:**
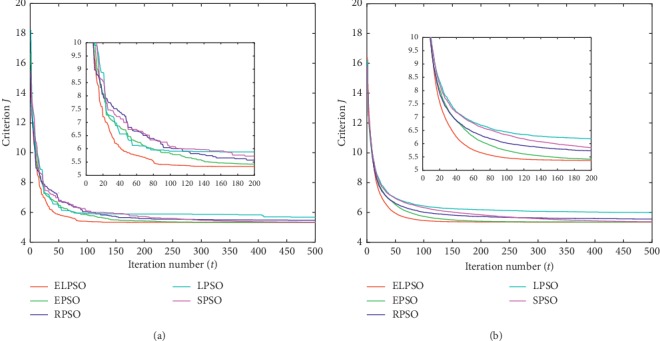
The clustering results of Ecoli dataset. (a) The best result in 30 times. (b) Average result in 30 times.

**Figure 3:**
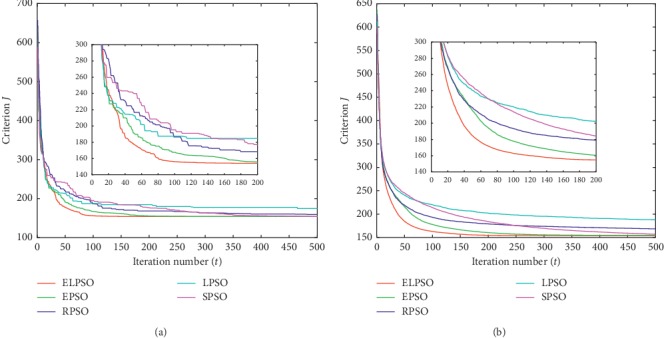
The clustering results of Glass dataset. (a) The best result in 30 times. (b) Average result in 30 times.

**Figure 4:**
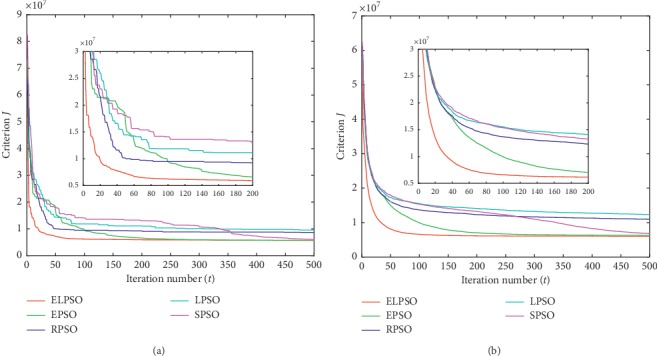
The clustering results of Image segmentation dataset. (a) The best result in 30 times. (b) Average result in 30 times.

According to the methodology used by Izakian and Abraham [16], criterion *J* is introduced to evaluate the clustering effect. The lower values of *J*, the better clustering effect could be obtained. Therefore, the experimental data with the minimum final value of criterion *J* were considered as the optimal result. The average value recorded was to account for the stochastic nature of the algorithms. For a better view of the results, the best values and the average values of *J* are shown in Figures 1–4, respectively.

Since the inertia weight plays an important role on the overall performance of the algorithm, in order to ensure that the variables are unique, all parameters are set consistently except the inertia weight. The parameter values for each algorithm are set as follows.

The population: all algorithms are set to 30 uniformly; ELPSO: *c*_1_ = *c*_2_ = 2, *ω* is dynamically adjusted according to the proposed strategy using equation (12), and *z* is set to 1.05; the parameters in other algorithms are set to be consistent with ELPSO and their inertial weight strategy reference literature [28–30].

The results are shown as follows.

For a better observation of experimental results, we extract the curves of the first 200 iterations separately and place them in the overall iteration graph. In this way, we can perceive the convergence trend of each algorithm explicitly. In addition, the criterion *J* of the 50^th^, 200^th^, and 500^th^ iterations is listed in Tables 2 and 3, respectively; these results represent the optimal value and average value in experiments.

Figures 1–4 show the result for the five approaches represented by five colored curves. In each figure, the horizontal axis represents the number of iterations, and the vertical axis represents criterion *J*. A smaller value of *J* indicates better results.

The optimal result in 30 iterations represents the extreme ability of algorithm, but the average result over 30 iterations can better illustrate the performance of algorithm. It is clearly seen from Figures 1to 4 that ELPSO converges more quickly and has obvious advantage of convergence speed in best graphs and average graphs than other algorithms, especially in the first fifty iterations.

Tables 2 and 3 show that ELPSO always achieves the smallest value for criterion *J* in 50^th^, 200^th^, and 500^th^ iterations, better than other four algorithms regardless of the best values or average values. Although in the Abalone dataset, EPSO finally obtained the same optimal value as ELPSO, but its earlier convergence rate was slower than ELSPO. From the results obtained from the four sets of datasets, LPSO is more likely to fall into a local optimum in five algorithms, and ELPSO has never been trapped in a local optimum due to its appropriate inertia weight selection strategy.

The results of the tests lead to the conclusion that the proposed ELPSO is efficient and has rapid convergence, can counterpoise the global search and local search more effectively, and can reveal very encouraging results in terms of quality of solution found.

### 4.2. Hybrid Methods Clustering

In this section, the FCM-ELPSO proposed in this work is compared to other four PSO-based hybrid algorithms which are FCM-SPSO, FCM-LPSO, FCM-EPSO, and FCM-RPSO. In addition, GA-FCM is added to the test. For evaluating the performance of all of the above algorithms, eight UCI datasets are selected: Ecoli, Glass, Image segmentation, Page blocks classification, Spectf, Steel plates faults, Ultrasonic flowmeter diagnostics, and Yeast, as shown in Table 1.

To quantitatively evaluate the convergence effect, the fundamental criterion can be described as follows: the distance between different objects in the same cluster should be as close as possible; the distance between different objects in different cluster should be as far as possible. The criterion *J* is still introduced to evaluate the clustering effect, as the same in Section 4.1. Additionally, an effective cluster validity index is introduced into the evaluation system, namely, PBM(F), which has been described in detail. It is worth reminding again that for a given dataset and the determined number of clusters, higher values of the PBM(F) index indicate better clustering in the sense that the clusters are well separated and relatively compact.

Each algorithm is run 30 times with random initializations for every dataset, and the partition that corresponds to the best criterion value is selected. Once the partition is selected, its corresponding PBM(F) is calculated. Furthermore, the average and standard deviation of the 30 repetitions are also computed for criterion *J* and validity index PBM(F). The parameters of the PSO part in these five algorithms are the same values as in Section 4.1, and the fuzzy parameter *m* in the FCM part is set to 2. The results are shown as follows.

Table 4 shows the best objective function values expressed in equation (4) obtained from the five clustering algorithms. For a more careful observation, the average values are provided separately in Table 5. It should be noted that the hybrid methods always converge before reaching the aforementioned maximum number of iterations [16]. Hence, it can be considered that under the same stopping condition, the performance of the algorithms depend on their results.

Tables 4 and 5 show that FCM-ELPSO always achieves the smallest value for criterion *J*. For further illustrating the performance of these algorithms, we introduce the standard deviation to describe the deviation degree of the mean values. The smaller the standard deviation value is, the smaller the convergence range is and the more robust the algorithm is. Table 4 shows the standard deviation for criterion *J*.

In Table 6, FCM-ELPSO gets the smallest standard deviation on five datasets, Glass, Page blocks classification, Spectf, Ultrasonic flowmeter diagnostics, and Yeast. FCM-SPSO gets two, Image segmentation and Steel plates faults, and FCM-LPSO has one, Ecoli. It can be seen that ELPSO has a smaller convergence range and higher robustness.

Tables 7–9 show the corresponding values for validity index PBM(F).

FCM-ELPSO gets the maximum on five datasets of best results for validity index PBM(F), as shown in Table 7, and FCM-RPSO performs better in Glass and Page blocks classification, while FCM-LPSO is good at Spectf. In terms of average results and standard deviation, FCM-ELPSO performs better than other algorithms. And it is noticed that the performance of GA-FCM is not as good as the hybrid clustering algorithms based on PSO.

Comparing the results of two cluster validity indexes, it is possible to notice that the best criterion *J* is not always associated with the best value for the PBM(F) because the cluster validity indexes are not applicable to all datasets. However, the experimental results can still prove that FCM-ELPSO performs better and has better robustness. The hybrid algorithm combines the merits of both algorithms to prevent premature convergence and trapping into local optimum effectively and improves the convergence speed slightly and obtains satisfactory results.

## 5. Conclusion

This paper proposes ELPSO to better balance between exploration and exploitation, which avoids falling into local optimum and has excellent convergence ability. In order to overcome the shortcomings of the PSO-based fuzzy clustering algorithms, ELPSO and FCM are combined to form a hybrid method called FCM-ELPSO, which utilizes the global search property of ELPSO to produce suitable initial clustering prototypes for FCM. FCM-ELPSO can correct the clustering direction during training constantly. So, as a randomized initialization approach, the hybrid method has the capability to alleviate the problems faced by FCM, which has some demerits of initialization and falling in local minima. The experiments test the ELPSO and the hybrid algorithm separately. Experimental results show that ELPSO and FCM-ELPSO perform well in the UCI datasets. In particular, FCM-ELPSO can produce higher quality clusters with a smaller standard deviation on the selected datasets compared with other clustering methods, especially in the high dimension and large data cases.

For future work, we will explore the practical application of the proposed methods in different fields, such as image segmentation, text mining, and medical problems. Furthermore, we will research novel initialization methods of PSO to improve the performance for complex datasets.

## Figures and Tables

**Algorithm 1:**
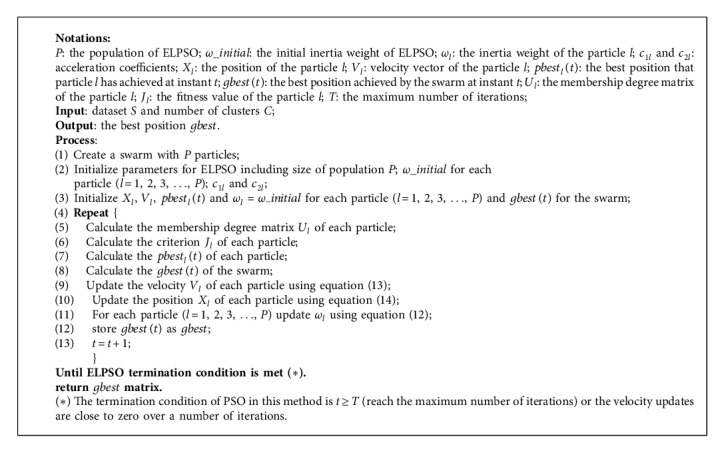
ELPSO clustering.

**Algorithm 2:**
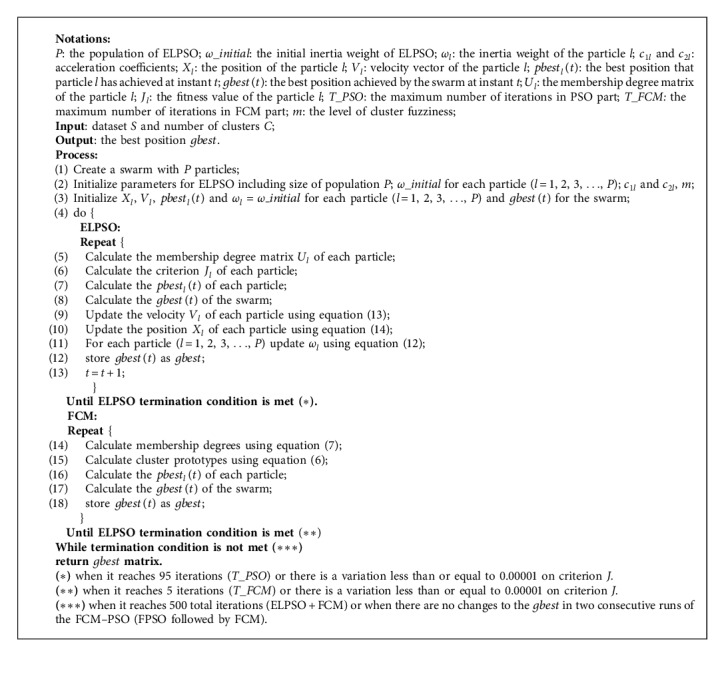
FCM-ELPSO.

**Table 1:** Descriptions of the real datasets.

Datasets	Objects	Variables	Groups
Abalone	4177	3	8
Ecoli	336	7	8
Glass	214	9	6
Image segmentation	2310	19	7
Page blocks classification	5473	10	5
Spectf	267	44	2
Steel plates faults	1941	27	7
Ultrasonic flowmeter diagnostics	361	43	4
Yeast	2000	8	10

**Table 2:** Best results for criterion *J* (the best results are highlighted in bold).

Datasets	Iterations	PSO	LPSO	EPSO	RPSO	ELPSO
Abalone	50	7610.0432	7928.2152	7321.0800	7456.6338	**7209.1766**
	200	7229.6062	7399.8084	7197.7556	7210.1214	**7197.7447**
	500	7197.7448	7237.2808	**7197.7447**	7198.3222	**7197.7447**
Ecoli	50	6.7325	6.3524	6.4713	6.8044	**5.8502**
	200	6.0905	5.9078	5.8227	6.0385	**5.3947**
	500	5.3443	5.6718	5.3314	5.4742	**5.3310**
Glass	50	240.1781	213.2250	191.1327	220.7926	**178.3978**
	200	176.6745	184.5977	155.7761	168.4756	**154.1854**
	500	154.5077	174.6182	154.1481	159.4427	**154.1460**
Image segmentation	50	18079261	14459116	16710175	10112452	**7263604**
	200	13038282	11080451	6606879	9258174	**5940593**
	500	6082020	9604572	5780101	8724927	**5690913**

**Table 3:** Average results for criterion *J* (best results are highlighted in bold).

Datasets	Iterations	PSO	LPSO	EPSO	RPSO	ELPSO
Abalone	50	7681.8732	7756.7018	7375.2852	7444.9952	**7210.9449**
	200	7224.8618	7380.1867	7197.7683	7211.3830	**7197.7447**
	500	7197.7452	7297.5891	**7197.7447**	7199.7810	**7197.7447**
Ecoli	50	6.9441	6.9788	6.5282	6.6143	**5.9576**
	200	5.8466	6.1840	5.4144	5.7297	**5.3656**
	500	5.3791	5.9953	5.3618	5.5613	**5.3537**
Glass	50	245.4625	240.3651	215.4950	219.1814	**184.6535**
	200	213.7898	220.0770	177.9124	193.2778	**162.8663**
	500	156.9724	187.9902	154.6609	168.6809	**154.1477**
Image segmentation	50	17945971	17274272	15048314	16121031	**8161111**
	200	13228454	14092457	7037767	12326004	**6154794**
	500	6811563	12371116	6298898	10979083	**6050846**

**Table 4:** Best results for criterion *J* (best results are highlighted in bold).

Datasets	GA-FCM	FCM-SPSO	FCM-LPSO	FCM-EPSO	FCM-RPSO	FCM-ELPSO
Ecoli	5.3561	5.3460	5.3490	5.3540	5.3457	**5.3326**
Glass	157.4681	155.3780	154.4778	155.1152	154.7951	**154.1496**
Image segmentation	6.0142*e* + 06	5.9676*e* + 06	5.8362*e* + 06	5.8933*e* + 06	5.8689*e* + 06	**5.7221*e* + 06**
Page blocks classification	8.5735*e* + 09	8.5614*e* + 09	8.5621*e* + 09	8.5643*e* + 09	8.5616*e* + 09	**8.5612*e* + 09**
Spectf	5.8436*e* + 05	5.8049*e* + 05	5.7739*e* + 05	5.7739*e* + 05	5.7739*e* + 05	**5.7739*e* + 05**
Steel plates faults	4.3874*e* + 14	4.2944*e* + 14	4.3438*e* + 14	4.3463*e* + 14	4.2936*e* + 14	**4.2900*e* + 14**
Ultrasonic flowmeter diagnostics	3.6411*e* + 08	3.6287*e* + 08	3.6312*e* + 08	3.6364*e* + 08	3.6310*e* + 08	**3.6276*e* + 08**
Yeast	12.2630	12.0382	11.8642	11.8538	11.8746	**11.8413**

**Table 5:** Average results for criterion *J* (best results are highlighted in bold).

Datasets	GA-FCM	FCM-SPSO	FCM-LPSO	FCM-EPSO	FCM-RPSO	FCM-ELPSO
Ecoli	5.4132	5.4074	5.3909	5.3943	5.3878	**5.3649**
Glass	160.6247	158.4809	158.7457	159.0322	159.6960	**154.9908**
Image segmentation	6.1894*e* + 06	6.0791*e* + 06	6.1117*e* + 06	6.1396*e* + 06	6.0897*e* + 06	**6.0510*e* + 06**
Page blocks classification	9.9203*e* + 09	9.8668*e* + 09	9.1186*e* + 09	9.3430*e* + 09	9.3317*e* + 09	**8.6631*e* + 09**
Spectf	5.8960*e* + 05	5.8260*e* + 05	5.7883*e* + 05	5.7838*e* + 05	5.7849*e* + 05	**5.7754*e* + 05**
Steel plates faults	4.5225*e* + 14	4.5225*e* + 14	4.8656*e* + 14	4.7123*e* + 14	4.6599*e* + 14	**4.4662*e* + 14**
Ultrasonic flowmeter diagnostics	3.6819*e* + 08	3.6763*e* + 08	3.6795*e* + 08	3.6904*e* + 08	3.6796*e* + 08	**3.6387*e* + 08**
Yeast	13.1546	12.1272	11.9522	11.9556	11.9585	**11.8478**

**Table 6:** Standard deviation for criterion *J* (best results are highlighted in bold).

Datasets	GA-FCM	FCM-SPSO	FCM-LPSO	FCM-EPSO	FCM-RPSO	FCM-ELPSO
Ecoli	0.0301	0.0297	**0.0223**	0.0245	0.0273	0.0272
Glass	2.8639	1.7319	2.4125	1.8354	2.0823	**1.1018**
Image segmentation	1.7584*e* + 05	**7.2663*e* + 04**	1.7584*e* + 05	1.4599*e* + 05	1.5925*e* + 05	2.7719*e* + 05
Page blocks classification	1.2927*e* + 09	1.3478*e* + 09	7.8227*e* + 08	1.4926*e* + 09	1.0365*e* + 09	**4.6222*e* + 08**
Spectf	1.4432*e* + 03	912.4835	1.9985*e* + 03	1.5790*e* + 03	1.6325*e* + 03	**261.1258**
Steel plates faults	3.6258*e* + 13	**1.7151*e* + 13**	3.8546*e* + 13	2.4850*e* + 13	3.4818*e* + 13	2.3890*e* + 13
Ultrasonic flowmeter diagnostics	3.9146*e* + 06	5.1001*e* + 06	3.4344*e* + 06	4.2497*e* + 06	3.7826*e* + 06	**1.0618*e* + 06**
Yeast	0.0472	0.0443	0.0627	0.0596	0.0705	**0.0065**

**Table 7:** Best results for validity index PBM(F) (best results are highlighted in bold).

Datasets	GA-FCM	FCM-SPSO	FCM-LPSO	FCM-EPSO	FCM-RPSO	FCM-ELPSO
Ecoli	0.3265	0.3274	0.3303	0.3308	0.3327	**0.3340**
Glass	3.0827	3.1856	3.7173	3.2463	**4.0843**	3.9848
Image segmentation	564.4213	566.3507	577.5760	573.2499	576.2624	**587.5084**
Page blocks classification	8.1091*e* + 04	8.5060*e* + 04	8.5086*e* + 04	7.7729*e* + 04	**8.7911*e* + 04**	8.3118*e* + 04
Spectf	26.4198	18.8760	**35.7040**	35.4333	33.3814	34.2975
Steel plates faults	1.0651*e* + 07	1.0634*e* + 07	1.0566*e* + 07	1.0686*e* + 07	1.0685*e* + 07	**1.0692*e* + 07**
Ultrasonic flowmeter diagnostics	4.0962*e* + 03	4.1664*e* + 03	4.0975*e* + 03	4.0582*e* + 03	4.1587*e* + 03	**4.2225*e* + 03**
Yeast	0.1082	0.1105	0.1395	0.1374	0.1378	**0.1526**

**Table 8:** Average results for validity index PBM(F) (best results are highlighted in bold).

Datasets	GA-FCM	FCM-SPSO	FCM-LPSO	FCM-EPSO	FCM-RPSO	FCM-ELPSO
Ecoli	0.3195	0.3162	0.3174	0.3198	0.3214	**0.3238**
Glass	2.6173	2.7959	2.9268	2.8091	2.9334	**3.3451**
Image segmentation	261.9715	255.1723	409.8047	401.2907	361.9401	**487.0102**
Page blocks classification	6.7247*e* + 04	6.8971*e* + 04	6.5584*e* + 04	6.7081*e* + 04	6.5419*e* + 04	**7.0711*e* + 04**
Spectf	19.5535	12.4250	26.0889	28.1726	27.2532	**31.5303**
Steel plates faults	9.6322*e* + 02	1.0076*e* + 07	9.5767*e* + 06	9.7646*e* + 06	9.7419*e* + 06	**1.0186*e* + 07**
Ultrasonic flowmeter diagnostics	3.6912*e* + 03	3.7835*e* + 03	3.7067*e* + 03	3.6883*e* + 03	3.7202*e* + 03	**4.0045*e* + 03**
Yeast	0.0871	0.0884	0.1207	0.1177	0.1191	**0.1434**

**Table 9:** Standard deviation for validity index PBM(F) (best results are highlighted in bold).

Datasets	GA-FCM	FCM-SPSO	FCM-LPSO	FCM-EPSO	FCM-RPSO	FCM-ELPSO
Ecoli	0.0085	0.0088	0.0060	0.0072	0.0065	**0.0053**
Glass	0.2473	0.1993	0.2953	0.2056	0.3306	**0.1647**
Image segmentation	200.4316	196.6843	207.5596	198.1861	216.7850	**168.7607**
Page blocks classification	7.1675*e* + 03	5.8166*e* + 03	6.7870*e* + 03	6.3222*e* + 03	7.4694*e* + 03	**2.8312*e* + 03**
Spectf	7.0519	2.9708	7.7021	6.6620	6.5179	**2.1903**
Steel plates faults	5.8661*e* + 05	4.2121*e* + 05	6.4348*e* + 05	5.2614*e* + 05	6.2299*e* + 05	**3.9786*e* + 05**
Ultrasonic flowmeter diagnostics	306.4910	331.9834	232.4805	245.6173	290.7265	**196.3196**
Yeast	0.0133	0.0107	0.0116	0.0129	0.0134	**0.0053**

## Data Availability

The data used to support the findings of this study are available from the corresponding author upon request.
